# Effects of Preoperative HbA1c on Postoperative Outcomes Following Transforaminal and Posterior Lumbar Interbody Fusion

**DOI:** 10.7759/cureus.106232

**Published:** 2026-03-31

**Authors:** Noah T Coleman, Ara Khoylyan, Matthew W Parry, Alex Tang, Tan Chen

**Affiliations:** 1 Orthopaedic Surgery, Geisinger Commonwealth School of Medicine, Scranton, USA; 2 Orthopaedic Surgery, Geisinger Health System, Wilkes-Barre, USA

**Keywords:** diabetes mellitus, hba1c, posterior lumbar interbody fusion (plif), promis score, transforaminal lumbar interbody fusion (tlif)

## Abstract

Introduction

Uncontrolled diabetes mellitus (DM) is associated with higher rates of postoperative complications and poor outcomes following spinal surgery. The purpose of this study was to compare postoperative patient-reported outcome measures (PROMs) between non-diabetic (non-DM) and diabetic (DM) patients undergoing posterior lumbar interbody fusion (PLIF) or transforaminal lumbar interbody fusion (TLIF), characterize the clinical trajectory and rate of improvement between non-DM and DM cohorts, and determine if a clinically relevant HbA1c cutoff exists.

Materials and methods

Retrospective analysis was performed, identifying non-DM and DM patients who underwent elective single or multilevel PLIFs or TLIFs for degenerative pathology between 2019 and 2023. Diabetes was defined as having a preoperative HbA1c ≥ 6.5%. Patient demographics, Oswestry disability index (ODI), and patient-reported outcomes measurement information system (PROMIS) scores were collected longitudinally. Maximum medical improvement (MMI) was defined as the time point where more than 90% of the cohort achieves minimal clinically important difference (MCID) for both ODI and PROMIS score reports. Descriptive and inferential statistics were performed.

Results

A total of 114 non-DM and 14 DM patients were included. The DM cohort was observed to have higher average BMI (non-DM: 30.7, DM: 35.0, p = 0.029, t = 2.210) and decreased availability of a care partner (non-DM: 80 (85%), DM: 6 (60%), p = 0.046, x^2 ^= 3.981). No difference was noted for cohort age (non-DM: 55.3 years, DM: 61.3 years, p = 0.097, t = 1.671), complications (non-DM: nine (8%), DM: zero (0%), p = 0.276, x^2 ^= 1.189), approach (non-DM minimally invasive surgery (MIS): nine (8%), non-DM open: 105 (92%), DM MIS: one (7%), DM open: 13 (93%), p = 0.921, x^2 ^= 0.010), procedure (non-DM TLIF: 29 (26%), non-DM PLIF: 84 (74%), DM TLIF: five (36%), DM PLIF: nine (64%), p = 0.423, x^2 ^= 0.624), or number of levels fused (non-DM: 1.3, DM: 1.1, p = 0.399, t = 0.845).

The DM cohort reported poorer preoperative PROMIS overall (non-DM: 28.6, DM: 23.7, p = 0.003, t = 3.071), PROMIS physical (non-DM: 37.5, DM: 33.1, p = 0.004, t = 2.904), and PROMIS mental (non-DM: 43.7, DM: 38.9, p = 0.023, t = 2.304) scores but were found to have similar preoperative ODI scores (non-DM: 45.2, DM: 53.1, p = 0.058, t = 1.912). Clinical improvement was comparable between the two groups, exceeding MCID at one-year follow-up. Both cohorts achieved MMI at similar rates.

Multivariate analysis showed that preoperative HbA1c was not correlated with the rate of MCID achievement, MMI, or outcome scores when controlling for age, BMI, sex, care partner presence, number of levels fused, procedure type, approach type, and complications. There was no difference in outcomes between MIS and open surgery patients.

Conclusion

Elevated preoperative HbA1c was correlated with worse preoperative outcome measures. Despite this, both diabetic and non-diabetic patients achieved similar postoperative recoveries following elective PLIF and TLIF, with no difference in long-term outcomes, complications, and rate of improvement. Our results highlight the importance of continued research on preoperative optimization, risk management, patient counseling, and the development of individualized treatment plans.

## Introduction

Degenerative spine disease (DSD) is a leading cause of chronic back pain [1]. As of 2018, it has been estimated that approximately 266 million cases of lumbar DSD with associated lower back pain occur worldwide each year [2]. Most commonly, DSD is initiated by a structural and functional loss within the intervertebral disc. External stress combined with internal degenerative changes in select populations leads to progressive mechanical loss and other structural failures [3]. Common etiologies of DSD include degenerative disc disease, disc herniation, spinal stenosis, spondylolisthesis, and myelopathy. Of these, it is estimated that 403 million individuals have disc-related degeneration, 103 million have spinal stenosis, and 39 million have spondylolisthesis worldwide [2]. Common etiologies include high-intensity sports, exercise, or strenuous repetitive daily activities, with family history, poor nutrition, current or former smoking history, age, comorbid conditions, and obesity as prominent risk factors [4]. Conservative measures are first-line treatment options and include anti-inflammatory medications, physical therapy, bracing, lifestyle modifications such as exercise and dietary changes, and steroid injections [5]. When these fail, surgery may be considered.

Transforaminal lumbar interbody fusion (TLIF) and posterior lumbar interbody fusion (PLIF) are commonly performed surgeries for degenerative conditions of the lumbar spine [6-8]. Indications may include disc-related low back pain, radiculopathy, foraminal stenosis, spondylolisthesis, and scoliosis [1]. Surgical intervention for DSD is associated with various complications, and prior research has demonstrated that preoperative health status is a major determinant of postoperative course and complication rate [9-14]. Diabetes mellitus (DM) is one such comorbidity that can increase the risk of peri- and postoperative adverse events [15,16]. DM continues to be one of the most common comorbidities worldwide [17]. As of January 2024, 9.7 million adults have undiagnosed diabetes, 29.3 million have a confirmed diagnosis, and 115.9 million adults have pre-diabetes [17]. Poorly controlled DM is well known to be associated with postoperative infection, transfusion, hematoma, pneumonia, in-hospital mortality, nonroutine discharge, respiratory distress, increased length of stay, and increased hospital charges following spinal surgery [18-20].

Previous studies have attempted to classify the significance of hemoglobin A1c (HbA1c), a measure of the average blood glucose over the past two to three months, on outcomes following spinal fusion; however, little consensus exists [21-23]. The meta-analysis performed by Tao et al. found elevated HbA1c to be associated with poorer postoperative clinical outcomes and surgical site infections [21]. These results are corroborated by Tanka et al., who found a higher incidence of post-surgical complications in those with poorly controlled DM [22]. However, Liow et al. discovered no difference in clinical scores or functional outcomes in diabetic and non-diabetic patients following cervical spinal fusions [23]. There is a lack of current research available to accurately assess individual fusion techniques and other surgical approaches, namely lumbar fusions. Given that, as of 2008, lumbar fusions are the most commonly performed fusions in the United States, with approximately 210,000 per year, understanding how and whether HbA1c influences lumbar fusions is important when considering fusion success rate, risk stratification, patient management, and tailored postoperative care [24]. For clinicians, it may be important to consider this when attempting to optimize patient satisfaction, control for clinical consequences, and advise patients on their recovery trajectories following surgical procedures.

The paucity of lumbar fusion data underscores the importance of current and future research, especially within comorbid populations. Given the increasing prevalence of diabetes and DSD, data focusing on the long-term outcomes associated with these diseases is essential to bring insight and awareness to this growing social problem. Both diabetes and DSD represent a major global financial burden. Diabetes-related health costs are expected to reach 1,045 billion US dollars by 2045 [25]. Similarly, the treatment of spinal disorders is estimated to cost more than 100 billion dollars per year [26]. To combat these collective burdens, further investigation would be beneficial in elucidating the impact of preoperative HbA1c and diabetic status on the postoperative clinical course following lumbar fusion. Specifically, the recovery rate following these types of procedures is not well investigated in prior literature.

In prior literature, the recovery rate following cervical fusion has been established by the maximum medical improvement (MMI), defined as the time point at which 90% of the population reaches minimal clinically important difference (MCID) between survey scores [27]. However, no prior studies have determined MMI following lumbar fusion. Utilizing patient-reported outcome measures (PROMs), including Oswestry disability index (ODI) and patient-reported outcome measurement information system (PROMIS) scores, the present study seeks to characterize postoperative outcomes in diabetic and non-diabetic patients based on PROM scores and complication rate. We also aim to determine the postoperative recovery trend and recovery rate based on MMI and MCID. Finally, we investigate whether there is a clinically relevant cutoff for preoperative HbA1c for determining outcomes [28,29].

This article was previously presented as a conference abstract at the International Society for the Advancement of Spine Surgery (ISASS) annual conference from April 10 to 12, 2025.

## Materials and methods

Exemption from the local institutional review board was obtained before data collection. A retrospective analysis was performed assessing patients who had an elective single- or multilevel TLIF or PLIF procedure between November 2019 and July 2023. Patients were initially identified using current procedural terminology (CPT) codes for TLIF (22633) and PLIF (22630). Patients at least 18 years of age who had a single or multilevel procedure for degenerative spine pathology with viable preoperative HbA1c levels within two weeks and completed pre- and postoperative PROM surveys met the inclusion criteria. Missing data for any collected dependent variable was not included within the overall results for the defined corresponding analysis. The minimum required follow-up time was 1.5 months. Patients with revision surgeries at the level of the index procedure were excluded. Manual chart review was performed, and data were collected in a longitudinal Excel chart for demographics, preoperative HbA1c level, PROM scores, surgery data (procedure type, surgical approach, and number of levels fused), postoperative care partner information (availability of a significant other, family member, or nursing aid for assistance with at-home care), and complications. Complications were defined as adverse events occurring after and were directly related to the surgical procedure that deviated from the expected recovery course. Intraoperative surgical standardization techniques were not accounted for.

Patient-reported outcome measures (PROMs)

The ODI and PROMIS global health, physical health, and mental health scores were obtained within one week preoperatively and again postoperatively at six weeks, 12 weeks, six months, and one year [30,31]. These scales and questionnaires are standardized and validated tools to assess subjective postoperative outcomes. They were incorporated into the online platform from which patients recorded their information at each defined time point and were approved for use. MCID was defined based on previous literature as a 10-point improvement in the ODI and an eight-point improvement in PROMIS scores in comparison to baseline preoperative score [32]. MMI was established as the time point at which 90% of the total population met MCID, based on prior literature similarly assessing recovery following cervical spine surgery.

Statistical analysis

Patients were divided into non-diabetic and diabetic cohorts based on preoperative HbA1c levels of <6.5% or ≥6.5%, as defined by the American Diabetes Association, obtained within two weeks before surgery [33]. Demographic data, follow-up time, surgical data (surgical approach, complications, and number of levels fused), and PROM scores were compared between the two groups using the Student’s t-test for continuous data and the chi-squared test for categorical data. Multivariate and univariate logistic regression analyses were completed between the preoperative HbA1c levels or diabetic status as the independent variables and PROM scores and differences in surgical outcomes as the dependent variables to determine the odds ratio (OR). Covariates in this analysis included age, sex, body mass index, and number of levels fused. A receiver operator characteristic (ROC) analysis was performed to determine the area under the curve (AUC) and determine clinically sensitive and specific thresholds using preoperative HbA1c as a predictor for postoperative outcomes. Preoperative HbA1c levels were compared with one-year postoperative PROMIS and ODI scores using Pearson’s correlation coefficient calculations. P-values ≤ 0.05 were determined to be statistically significant. All statistical calculations were completed using SPSS v30 (IBM Corp., Armonk, NY).

## Results

**Figure 1:**
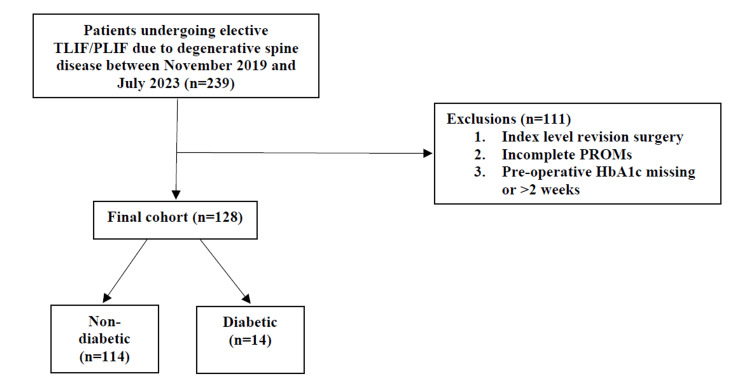
Determination of final patient cohorts based on eligibility criteria PLIF: Posterior lumbar interbody fusion; TLIF: Transforaminal lumbar interbody fusion; PROMs: Patient-reported outcome measures.

Of 327 patients identified undergoing elective TLIF/PLIF due to DSD, a total of 128 patients met the inclusion criteria for the study (Figure 1).

The population had a mean age of 56.0 ± 12.8 years and a mean BMI of 31.0 ± 5.8 kg/m^2^. Females had a greater prevalence within the distribution compared to males. PLIF was the approach most often used by surgeons, followed by TLIF. The minimally invasive surgery (MIS) approach was seldom used compared to its open counterpart. Single-level fusions were most prevalent within the distribution, followed by two-level and three-level (Table 1).

**Table 1:** Descriptive information of the total patient population BMI: Body mass index; MIS: Minimally invasive surgery; TLIF: Transforaminal lumbar interbody fusion; PLIF: Posterior lumbar interbody fusion.

N	128
Age	56.0 ± 12.8
BMI	31.0 ± 5.8
Sex	
Female	74 (58%)
Male	54 (42%)
Preoperative HbA1c (%)	5.7 ± 0.7
Diabetic status	
Diabetic (HbA1c ≥ 6.5%)	14 (11%)
Non-diabetic (HbA1c < 6.5%)	114 (89%)
Postoperative complications	
Yes	9 (7%)
No	119 (93%)
Postoperative care partner	
Yes	86 (83%)
No	18 (17%)
Approach	
MIS	10 (8%)
Open	118 (92%)
Procedure	
TLIF	34 (27%)
PLIF	93 (73%)
Fusion level(s)	
Single	99 (77%)
Multiple	29 (23%)
Number of levels fused	
1	99 (77%)
2	27 (21%)
3	2 (2%)
Mean number of levels fused	1.2 ± 0.5

Overall, peri- or postoperative complications were not common, occurring in only nine (7.9%) patients. Of these, the majority included dural tears and those requiring revision surgery, including adjacent segment disease, adjacent facet fracture, and post-laminectomy syndrome. Surgical wound dehiscence occupied a small minority of cases (Table 2).

**Table 2:** Distribution of peri- and postoperative complications based on diabetic status BMI: Body mass index; MIS: Minimally invasive surgery; TLIF: Transforaminal lumbar interbody fusion; PLIF: Posterior lumbar interbody fusion.

Complication	Total (N = 128)	Non-DM (N = 114)	DM (N = 14)
Complications (Total)	9	9 (7.9%)	0 (0%)
Dural tear	4	4 (3.5%)	0 (0%)
Revision surgery	4	4 (3.5%)	0 (0%)
Adjacent segment disease	1	1 (0.9%)	0 (0%)
Post-laminectomy syndrome	2	2 (1.7%)	0 (0%)
Adjacent facet fracture	1	1 (0.9%)	0 (0%)
Surgical wound dehiscence	1	1 (0.9%)	0 (0%)

A total of 114 (89%) patients were categorized as non-DM and 14 (11%) as DM. Between both groups, the mean BMI met the clinical classification of obese. However, the DM cohort had a higher BMI compared to the non-DM group (p = 0.029, t = 2.210). Additionally, the DM cohort had a lower rate of access to a postoperative care partner (p = 0.046, x^2 ^= 3.981). There were no significant differences in age (p = 0.097, t = 1.671), sex (p = 0.096, x^2 ^= 2.777), peri- or postoperative complications (p = 0.276, x^2 ^= 1.189), surgical approach (p = 0.921, x^2 ^= 0.010), procedure type (p = 0.423, x^2 ^= 0.642), or fusion type (p = 0.428, x^2 ^= 0.629) between the two cohorts (Table 3). Similarly, the category of levels fused did not differ (p = 0.693, x^2 ^= 0.733), nor did the average number of levels fused (p = 0.399, t = 0.845) (Table 3).

**Table 3:** Analysis of descriptive characteristics comparing diabetic and non-diabetic patient cohorts P-values calculated using Student’s t-test and chi-square test. BMI: Body mass index; MIS: Minimally invasive surgery; TLIF: Transforaminal lumbar interbody fusion; PLIF: Posterior lumbar interbody fusion.

	Non-diabetic (A1c < 6.5), N = 114	Diabetic (A1c ≥ 6.5), N =14	p-value	t-value	x^2^-value
Age	55.3 ± 12.7	61.3 ± 12.8	0.097	1.671	
BMI	30.7 ± 5.9	35.0 ± 3.2	0.029	2.210	
Sex					
Female	63 (55%)	11 (79%)	0.096		2.777
Male	51 (45%)	3 (21%)			
Postoperative complications					
Yes	9 (8%)	0 (0%)	0.276		1.189
No	105 (92%)	14 (100%)			
Postoperative care partner					
Yes	80 (85%)	6 (60%)	0.046		3.981
No	14 (15%)	4 (40%)			
Approach					
MIS	9 (8%)	1 (7%)	0.921		0.010
Open	105 (92%)	13 (93%)			
Procedure					
TLIF	29 (26%)	5 (36%)	0.423		0.642
PLIF	84 (74%)	9 (64%)			
Fusion(s)					
Single	87 (76%)	12 (86%)	0.428		0.629
Multiple	27 (24%)	2 (14%)			
Number of levels fused					
1	87 (76%)	12 (86%)	0.693		0.733
2	25 (22%)	2 (14%)			
3	2 (2%)	0 (0%)			
Mean number of levels fused	1.3 ± 0.5	1.1 ± 0.4	0.399	0.845	

The DM cohort had no significant differences in mean ODI scores preoperatively or postoperatively. However, the DM cohort had significantly poorer preoperative PROMIS overall (p = 0.003, t = 3.071), PROMIS physical (p = 0.004, t = 2.904), and PROMIS mental (p = 0.023, t = 2.304) scores. DM was similarly associated with worse scores at six-month postoperative PROMIS overall (p = 0.040, t = 2.087), and PROMIS physical (p = 0.007, t = 2.738) (Table 4).

**Table 4:** Comparing postoperative survey scores at each time point between diabetic and non-diabetic patients P-value calculated using the Student’s t-test. ODI: Oswestry Disability Index; PROMIS: Patient-reported outcomes measurement information system.

	Timepoint	Non-diabetic (A1c < 6.5), N = 114	Diabetic (A1c ≥ 6.5), N = 14	p-value	t-value
ODI	Pre-op	45.2 ± 14.4	53.1 ± 17.4	0.058	1.912
	6 weeks	36.3 ± 13.5	31.5 ± 9.7	0.497	0.686
	12 weeks	29.0 ± 17.8	28.3 ± 14.6	0.895	0.132
	6 months	24.0 ± 17.6	31.8 ± 18.4	0.189	1.323
	1 year	23.6 ± 18.5	27.5 ± 20.3	0.518	0.649
PROMIS overall	Pre-op	28.6 ± 5.7	23.7 ± 5.0	0.003	3.071
	6 weeks	31.6 ± 5.2	31.5 ± 6.5	0.973	0.034
	12 weeks	33.0 ± 7.2	30.4 ± 5.1	0.206	1.273
	6 months	34.0 ± 7.7	28.6 ± 7.5	0.040	2.087
	1 year	33.5 ± 8.5	31.6 ± 8.5	0.481	0.708
PROMIS physical	Pre-op	37.5 ± 5.5	33.1 ± 4.4	0.004	2.904
	6 weeks	42.5 ± 5.1	41.2 ± 5.3	0.619	0.501
	12 weeks	44.6 ± 7.7	41.7 ± 5.6	0.182	1.344
	6 months	46.1 ± 8.1	38.9 ± 6.5	0.007	2.738
	1 year	45.2 ± 9.2	42.1 ± 9.1	0.299	1.044
PROMIS mental	Pre-op	43.7 ± 7.4	38.9 ± 6.1	0.023	2.304
	6 weeks	46.3 ± 7.0	45.9 ± 8.2	0.914	0.109
	12 weeks	46.4 ± 9.1	44.3 ± 5.4	0.410	0.827
	6 months	47.0 ± 9.8	42.5 ± 9.7	0.172	1.377
	1 year	47.1 ± 10.1	46.2 ± 9.9	0.791	0.266

There were no differences in one-year postoperative MCID achievement rate between the two cohorts for ODI (p = 0.554, x^2 ^= 0.350), PROMIS overall (p = 0.934, x^2 ^= 0.007), PROMIS physical (p = 0.503, x^2 ^= 0.449), or PROMIS mental (p = 0.988, x^2 ^= 2.257e-04) (Table 5).

**Table 5:** Comparison of the rate of achieving MCID based on scores at the end of the survey period (one year). MCID is defined as a decrease of 10 points in the ODI score or an increase of 8 points in the PROMIS score. P-value calculated using the chi-square test. MCID: Minimal clinically important difference; ODI: Oswestry Disability Index; PROMIS: Patient-reported outcomes measurement information system.

MCID One-Year		Non-diabetic (A1c < 6.5)	Diabetic (A1c ≥ 6.5)	p-value	x^2^-value
ODI	Not met	23 (26%)	2 (18%)	0.554	0.350
	Met	64 (74%)	9 (82%)		
PROMIS overall	Not met	53 (62%)	7 (64%)	0.934	0.007
	Met	32 (38%)	4 (36%)		
PROMIS physical	Not met	45 (53%)	7 (64%)	0.503	0.449
	Met	40 (47%)	4 (36%)		
PROMIS mental	Not met	62 (73%)	8 (73%)	0.988	2.257e-04
	Met	23 (27%)	3 (27%)		

Similarly, there was no difference in the number of patients who achieved MCID at any postoperative time point within each PROM score or between the two cohorts (Table 6).

**Table 6:** Determining differences in MMI based on the cumulative proportion of the patient cohort that met the MCID by each time point P-value calculated using the chi-square test. MCID: Minimal clinically important difference; ODI: Oswestry Disability Index; PROMIS: Patient-reported outcomes measurement information system.

Timepoint MCID Met		Non-diabetic (A1c < 6.5), N = 112	Diabetic (A1c ≥ 6.5), N = 14	p-value	x^2^-value
ODI	6 weeks	16/37 (43%)	2/4 (50%)	0.796	0.067
	12 weeks	70/102 (69%)	11/14 (79%)	0.447	0.578
	6 months	82/107 (77%)	11/14 (79%)	0.872	0.026
	1 year	92/112 (82%)	11/14 (79%)	0.744	0.106
PROMIS overall	6 weeks	7/37 (19%)	2/4 (50%)	0.154	2.035
	12 weeks	28/100 (28%)	7/14 (50%)	0.095	2.974
	6 months	43/105 (41%)	7/14 (50%)	0.519	0.415
	1 year	50/112 (45%)	8/14 (57%)	0.376	0.783
PROMIS physical	6 weeks	10/37 (27%)	0/4 (0%)	0.232	1.430
	12 weeks	45/98 (46%)	6/14 (43%)	0.830	0.046
	6 months	60/105 (57%)	6/14 (43%)	0.312	1.021
	1 year	67/112 (60%)	7/14 (50%)	0.482	0.495
PROMIS mental	6 weeks	7/37 (19%)	0/4 (0%)	0.339	0.913
	12 weeks	19/100 (19%)	2/14 (14%)	0.670	0.182
	6 months	29/105 (28%)	3/14 (21%)	0.624	0.241
	1 year	35/112 (31%)	4/14 (29%)	0.838	0.042

There was no significant correlation between preoperative HbA1c levels and one-year postoperative PROM scores (Table 7).

**Table 7:** Correlation between preoperative HbA1c level and one-year postoperative survey score P-value calculated using correlation analysis. R: Pearson correlation coefficient; ODI: Oswestry Disability Index; PROMIS: Patient-reported outcomes measurement information system.

	Total		Non-Diabetic		Diabetic	
One-Year Score	R	P-value	R	P-value	R	p-value
ODI	0.089	0.384	0.152	0.159	-0.346	0.297
PROMIS overall	-0.054	0.603	-0.009	0.935	0.096	0.778
PROMIS physical	-0.104	0.314	-0.086	0.436	0.240	0.477
PROMIS mental	0.007	0.949	0.071	0.517	-0.051	0.882

**Figure 2:**
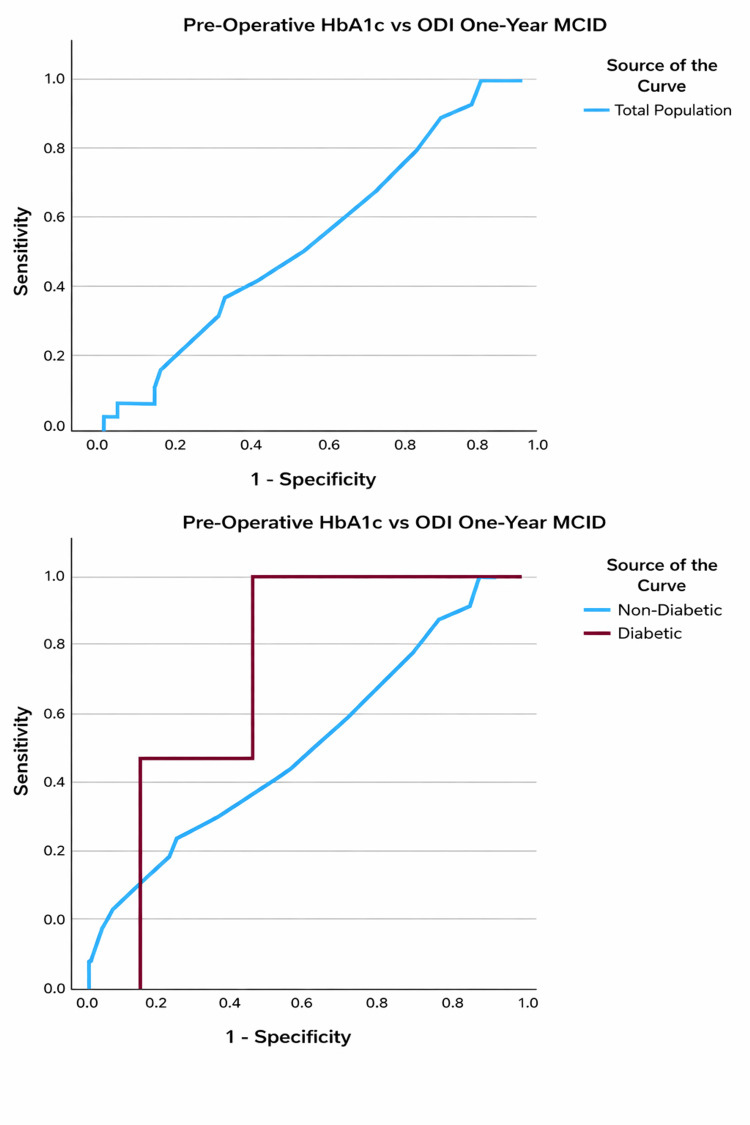
ROC curves evaluating predictive capability of preoperative HbA1c for failure to meet ODI MCID at the one-year postoperative time point in the total, non-diabetic, and diabetic populations ODI: Oswestry disability index; MCID: Minimal clinically important difference.

On ROC subanalysis of the diabetic cohort, a preoperative HbA1c level of ≥7.2% demonstrated 100% sensitivity and 67% specificity of detecting failure to achieve one-year postoperative ODI MCID (AUC = 0.778, p = 0.039) (Table 8 and Figure 2).

**Table 8:** ROC curve analysis of optimal preoperative HbA1c thresholds for determining the risk of not achieving one-year MCID based on ODI and PROMIS survey types P-value calculated using ROC curve analysis. ROC: Receiver operating characteristic; AUC: Area under the curve; Sens: Sensitivity; Spec: Specificity; MCID: Minimal clinically important difference; ODI: Oswestry Disability Index; PROMIS: Patient-reported outcomes measurement information system.

	Total					Non-diabetic (A1c < 6.5)					Diabetic (A1c ≥ 6.5)				
	AUC	P	HbA1c Cutoff	Sens	Spec	AUC	P-value	HbA1c Cutoff	Sens	Spec	AUC	P-value	HbA1c Cutoff	Sens	Spec
ODI MCID (one year)	0.543	0.515	5.15	0.92	0.12	0.577	0.147	5.15	0.90	0.14	0.778	0.039	7.15	1.00	0.67
			5.85	0.28	0.78			5.95	0.21	0.90			7.80	0.50	0.90
PROMIS overall MCID (one year)	0.472	0.663	5.15	0.92	0.11	0.534	0.616	4.95	0.91	0.04	0.768	0.092	6.60	1.00	0.29
			6.40	0.12	0.89			5.95	0.16	0.87			7.30	0.75	0.86
PROMIS physical MCID (one year)	0.513	0.822	5.15	0.89	0.11	0.507	0.916	5.05	0.93	0.09	0.768	0.092	6.60	1.00	0.29
			6.40	0.14	0.91			5.45	0.65	0.60			7.30	0.75	0.86
PROMIS mental MCID (one year)	0.495	0.930	5.15	0.91	0.17	0.507	0.906	5.15	0.90	0.19	0.573	0.646	6.60	0.89	0.17
			5.95	0.24	0.76			5.95	0.16	0.86			7.05	0.63	0.50

**Figure 3:**
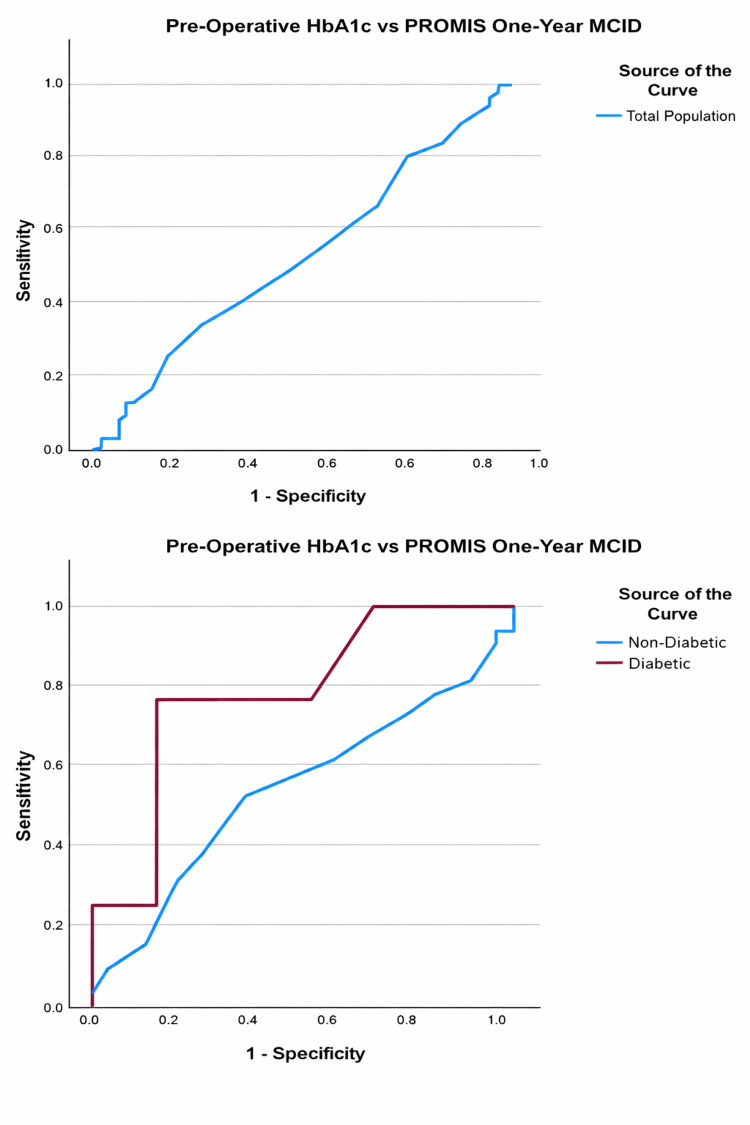
ROC curves evaluating predictive capability of preoperative HbA1c for failure to meet PROMIS MCID at the one-year postoperative time point in the total, non-diabetic, and diabetic populations PROMIS: Patient-reported outcomes measurement information system; MCID: Minimal clinically important difference.

Similarly, a preoperative value of ≥7.3% demonstrated 75% sensitivity and 86% specificity in detecting failure to achieve one-year postoperative PROMIS overall and physical MCIDs (AUC = 0.768, p = 0.092) (Table 8 and Figure 3). Preoperative HbA1c was a poor predictor of meeting ODI and PROMIS MCID in non-DM patients (Table 8 and Figures 2, 3).

## Discussion

DM is a well-known condition that can negatively influence the peri- and postoperative course [15,16]. Prior studies on the impact of HbA1c on postoperative MCID achievement are sparse, particularly for lumbar fusion. Our study evaluates the predictive capability of HbA1c on MCID achievement based on subjective PROM scores. Additionally, we sought to evaluate the effect of diabetic status on postoperative recovery rate with MMI, which has not been previously investigated in the setting of lumbar fusion surgery and, as such, has no documented maximal recovery threshold. Therefore, we expand on prior literature by comparing the long-term clinical trajectory between the two patient populations.

In patients with DM, preoperative HbA1c has a strong predictive capability for failure to achieve MCID based on ODI and PROMIS scores at one year following TLIF or PLIF surgery. We found a significant threshold at 7.2% (Sn: 100%, Sp: 67%) for ODI and 7.3% (Sn: 75%, Sp: 86%) for PROMIS surveys. These findings are in line with prior research suggesting DM patients with higher preoperative HbA1c levels have a greater risk of not achieving meaningful outcomes following surgery and emphasize the role of glycemic control in this population [34-36]. Gupta et al. similarly investigated these outcomes in diabetic patients following lumbar decompression and identified a preoperative HbA1c threshold of 7.5% (AUC = 0.65, p = 0.001) to be associated with a decreased likelihood of achieving MCID for ODI and 7.8% for NRS back pain (AUC = 0.65, p < 0.001) [37]. Similarly, other studies have indicated that patients with a preoperative HbA1c > 8% achieve lower rates of functional improvement post-lumbar surgery in an analysis of multiple spine surgical techniques, including fusion (RR: 1.85; 95% CI: 1.48-2.31; p < 0.01) (OR: 0.64; 95% CI: 0.44-0.92; p = 0.016) [21,38].

Based on this study’s results, diabetic patients undergoing TLIF or PLIF may benefit from discussion about the impact of their preoperative HbA1c level on long-term functional recovery following surgery, with levels > 7.2% being highly predictive of failure to meet recovery goals by one year. Similarly, discussion regarding perioperative diabetic management may also help patients with their surgical course. Specifically, a targeted blood glucose range of 80-180 mg/dl as recommended by the American Diabetes Association (ADA), optimal management of related complications, and adjusting the medication regimen based on blood glucose, nutritional status, and expected clinical status changes may improve outcomes [39]. Furthermore, given that current literature largely focuses in similar approach on recovery following cervical fusion, these findings can help provide more contextual insight about the trajectory after lumbar fusion [23,40,41].

Additionally, our study demonstrates that preoperative HbA1c level is not a sufficient predictor of postoperative functional recovery based on PROMs in non-DM patients, including prediabetics. Prior research has attempted to establish a predictive value for HbA1c in non-DM patients with little consensus [42,43]. Karimian et al. performed a systematic review of elevated preoperative HbA1c values in non-DM patients and their association with postoperative complications of multiple surgical modalities. Two studies reported higher postoperative infection rates in those with elevated HbA1c, while two others reported no difference. Of the four studies that examined hospital stay, only one supported an elevation in HbA1c to be indicative of longer stays.Only one study was associated with a higher postoperative mortality rate in patients with suboptimal HbA1c [42]. Similarly, Wang et al. investigated the effect of preoperative HbA1c levels on postoperative outcomes of coronary artery disease surgery in DM and non-DM patients, finding that higher preoperative HbA1c levels may potentially increase the risk of mortality and renal failure in non-DM patients (OR: 2.23, 95% CI: 1.01-4.90) (OR: 2.33, 95% CI: 1.32-4.12) [43]. The sum of these findings suggests that while preoperative HbA1c may be predictive of outcomes in non-DM patients following certain procedures, its utility is questionable for non-DM in the setting of lumbar fusion. Other factors such as demographic characteristics, comorbidities, lifestyle, socioeconomic status, genetic predisposition, level of health literacy, access to peri- or postoperative care, or surgical technique should deserve stronger consideration in this population.

While DM patients had worse preoperative PROMIS scores, no significant differences were observed in ODI scores or PROM scores one year following lumbar fusion. In 2021, Nagata et al. reported that DM patients have worse overall PROM scores one year following lumbar spinal surgery, though their investigation included a significantly larger sample size of 152 diabetic patients undergoing fusion [44]. Conversely, Thever et al. published a study consisting of 30 diabetic patients undergoing minimally invasive TLIF and found no differences in ODI scores two years post-operation (p = 0.512), which is more in line with our findings [45]. While the present study’s sample size of 14 DM patients is relatively small, the findings do suggest that even in the case of open TLIF, DM patients recover similarly with reference to functional status, despite, on average, being significantly older and having decreased access to a postoperative care partner. In fact, despite the small sample size, we were able to detect significantly poorer preoperative PROM scores in the diabetics, despite the relatively equal scores at one year following operation. It is possible that following patients longer than one year after the operation may reveal differences in PROM scores and recovery status. Such studies involving larger cohorts may be warranted to assess the durability of surgical outcomes, identify patterns of readmission or reintervention, monitor disease progression in high-risk groups, and further power or refute our results.

These findings are further corroborated by the fact that there was no difference in MMI between the non-DM and DM patients up to one year following surgery. While prior literature cites the target of 90% for determining MMI following cervical spine surgery, neither of our lumbar fusion cohorts reached this goal, demonstrating an overall slower recovery trend following lumbar fusion compared to cervical fusion [27]. To the authors’ knowledge, no prior studies have examined MMI in the context of lumbar fusion, and our findings suggest that either a lower threshold or longer postoperative follow-up with PROMs should be established for the detection of MMI. By one year, 92 (82%) non-DM and 11 (79%) DM cohorts achieved ODI MCID (p = 0.744, x^2 ^= 0.106), 50 (45%) and 8 (57%) achieved PROMIS overall MCID (p = 0.376, x^2 ^= 0.783), 67 (60%) and 7 (50%) achieved PROMIS physical MCID (p = 0.482, x^2 ^= 0.495), and 35 (31%) and 4 (29%) achieved PROMIS mental MCID (p = 0.838, x^2 ^= 0.042), demonstrating an overall similar rate of recovery.It is well known that optimal diabetic management during the operative period is essential to reduce poor postoperative outcomes, though advancements in insulin administration, continuous glucose monitoring, rehabilitation technology, and perioperative medicinal management have helped facilitate better outcomes over time [39,46,47]. We hypothesize that effective care teams employing these techniques with up-to-date guidelines explain the parity in this study’s cohorts and help mitigate known healing differences in diabetic patients. Similarly, the disparity between our results and known differences in diabetic healing may reflect our small sample size. Nevertheless, these findings can better guide anticipation of MMI in non-DM and DM patients undergoing TLIF or PLIF surgery.

It should also be noted that individual expectations, pain tolerance, and perceived disease burden are highly subjective, can influence PROM score, and may not differ significantly between DM and non-DM patients [48]. Additionally, the threshold for spinal surgical care is recommended at <7.5% preoperative HbA1c according to the Congress of Neurological Surgeons [49]. This study’s institutional health plan supports the guidelines set by the ADA standards of care, recommending HbA1c < 8%, which was also a limitation set in the study by Thever et al. [50]. While our findings do not make a case for delaying surgical care, the proposed preoperative HbA1c threshold of 7.2% is in line with guidelines and further supports the utility of preoperative evaluation of HbA1c not only for immediate surgical planning but also for postoperative decision-making and goal-setting for diabetic patients planning to undergo lumbar fusion. Overall, while further research with a larger diabetic cohort is warranted to conclusively demonstrate the impact of diabetic status on recovery following TLIF/PLIF, this study’s findings, in addition to prior literature, suggest that recovery is unaffected by diabetic status with both open and MIS techniques.

Finally, we found no statistically significant relationship between preoperative HbA1c levels and peri- or postoperative complications. However, the impact of preoperative diabetic status on predicting postoperative complication rate is well known, and multiple studies have demonstrated this association [18,20,51]. Arrighi-Allisan et al. discovered that diabetic patients were more likely to experience postoperative acute kidney injury, myocardial infarction, cardiac arrest, and pneumonia following posterior lumbar fusion [51]. Likewise, diabetic patients were found to have increased postoperative ICU stay and non-home discharges, prolonged length of stay, and an increased 30- and 90-day readmissions and emergency room visits [51]. In addition to this, diabetic patients accrued an average of $2834.00 increase in costs following surgery. Browne et al. also found an increase in pneumonia, nonroutine discharge, postoperative infection, and transfusion of diabetic patients following spinal fusions (p < 0.002) [18]. Similarly, Ruggiero et al. showed diabetics to have increased postoperative pulmonary and renal complications, surgical site infection, and prolonged length of stay following cervical and lumbar fusions [20]. There were only nine total reported complications in our study, all within the non-DM cohort. Of these, four were intraoperative dural tears; four were revision surgeries for adjacent segment disease, post-laminectomy syndrome, and adjacent facet fracture; and one was surgical wound dehiscence. Therefore, the sample size of this study was likely too small to adequately power for the detection of differences in postoperative complications between the two cohorts. Other prior studies, such as those by Ruggierio and Arrighi-Allisan et al., compiled data from larger national databases and explored different fusion types. Given that it is largely agreed upon that patients with diabetes carry a higher risk of either a medical or surgical complication following elective spinal surgery, our findings are not sufficient to refute prior research on this topic [52-54].

Limitations

Our study has several limitations that should be considered. First, the retrospective design limited the availability of preoperative HbA1c values and completed PROMs, reducing our sample size and subjecting the results to selection bias. The reduced sample size limits our statistical power and increases the risk of a Type II error while also compromising generalizability. Though this limitation is typical for studies of this nature, it may compromise the impact of our conclusions on the current scientific literature.

Our data were collected from a single institutional center in a rural setting, further limiting generalizability to other demographics. Likewise, the inclusion of patients with preoperative HbA1c values > 8% was limited due to institutional protocols that are nearly ubiquitous in elective spine surgery. Additionally, the reliance on subjective score reports and the lack of objective surgical outcomes could have potentially introduced recall bias and limited the power of our conclusions regarding recovery. We did not stratify diabetic severity, type, or duration, which may reveal intracohort differences that we were unable to detect. Confounding variables such as other comorbidities, medication adherence or regimen, additional therapeutic measures, and socioeconomic status were not controlled for and could have influenced the results. Similarly, the intergroup discrepancy in cohort sizes may also confound our results, as well as BMI differences and the availability of a care partner​​​​​​. PROM data were only collected up to one year following the operation, which may not capture the full recovery trend of all patients, especially in the long term. Lastly, our study does not account for other surgical variables or standardize surgical protocols such as intraoperative technique, cage material, bone graft, plate, or ceramic usage in comparison to postoperative outcomes, which may limit reproducibility.

## Conclusions

Diabetic patients undergoing elective TLIF or PLIF stand to benefit from measurement of preoperative HbA1c for long-term postoperative planning. Preoperative HbA1c is not a reliable predictor of meaningful clinical improvement in non- and prediabetic populations, suggesting that other factors can effectively mitigate the impact of diabetic status on postoperative recovery rate following TLIF and PLIF surgeries. These conclusions may be limited in generalizability given this study's small case sample size. Future research on lumbar spinal fusions in diabetic populations is essential due to the growing evidence linking diabetes with musculoskeletal diseases, post-surgical complications, and the increasing prevalence within the population. Further investigation into MMI thresholds and longer postoperative follow-up with PROMs concerning lumbar-specific fusions would benefit in characterizing optimal management in diabetic patients undergoing spinal surgery. Likewise, comparing diabetic status and postoperative course to other patient-specific variables such as health literacy, genetics, other comorbidities, or socioeconomic status may better demonstrate the steps necessary for a proper care plan for diabetic patients following surgical procedures. Such studies may lead to improved diagnostic tools, specific targeted interventions, preventative strategies, and improved standardized guidelines tailored to this high-risk population.
